# Combination of Cu-BTC- and FeCo-MOF-Derived Carbon Enhanced Molecularly Imprinted Electrochemical Sensor for Highly Sensitive and Selective Detection of Benomyl in Fruits and Vegetables

**DOI:** 10.3390/molecules30091869

**Published:** 2025-04-22

**Authors:** Lili Chen, Shuya Xue, Xin Li, Linbo Deng, Jiapeng Li, Jing Zhou, Yansha Gao, Xuemin Duan, Limin Lu

**Affiliations:** 1 Flexible Electronics Innovation Institute (FEII), Jiangxi Science and Technology Normal University, Nanchang 330013, China; 19314658155@163.com (L.C.); xueshuya0612@163.com (S.X.); 13133270915@163.com (J.L.); 2 Key Laboratory of Chemical Utilization of Plant Resources of Nanchang, College of Chemistry and Materials Sciencee, Jiangxi Agricultural University, Nanchang 330045, China; 18370786250@163.com (X.L.); a845831015@163.com (L.D.); von079@126.com (J.Z.); 3 Ji’an Key Laboratory of Photoelectric Crystal Materials and Device, Key Laboratory of Jiangxi Province for Special Optoelectronic Artificial Crystal Materials, School of Chemistry and Chemical Engineering, Humic Acid Utilization Engineering Research Center of Jiangxi Province, Institute of Applied Chemistry, Jinggangshan University, Ji’an 343009, China

**Keywords:** MOF-derived carbon, Cu-BTC, pesticide detection, benomyl, electrochemical sensor

## Abstract

The development of sensitive and selective methods for detecting pesticide residues has become paramount for ensuring food safety. In this work, a high-performance molecularly imprinted electrochemical sensor based on the composite of Cu-BTC- and FeCo-ZIF-derived N-doped carbon (FeCo@NC), synthesized by pyrolysis and electrodeposition, was developed for Benomyl (BN) detection. The materials were characterized by scanning electron microscopy (SEM), X-ray diffraction (XRD), and X-ray photoelectron spectroscopy (XPS). In this sensing system, the Cu-BTC/FeCo@NC composite used as the electrode substrate displayed a large specific surface area, high electronic conductivity, and rich active catalytic sites, demonstrating excellent electrocatalytic ability toward BN oxidation. Meanwhile, Cu-BTC, with its abundant surface functional groups, facilitated strong hydrogen bonding interactions with the imprinted template molecule of 3,4-ethylenedioxythiophene (EDOT), promoting the formation of a uniform molecularly imprinted membrane on the substrate material surface. The introduced MIP-PEDOT could enhance the selective recognition and enrichment of the target BN, leading to an amplified detection signal. Thanks to the synergistic effects between Cu-BTC/FeCo@NC and MIP-PEDOT, the proposed sensor achieved a low detection limit of 1.67 nM. Furthermore, the fabricated sensor exhibited high selectivity, reproducibility, and interference resistance in detecting BN. The method has been successfully applied to the determination of BN in vegetable and fruit samples, indicating its potential for use in practical applications.

## 1. Introduction

Benomyl (BN), a benzimidazole pesticide and broad-spectrum insecticide, is widely used to prevent and control pests in fruits and vegetables, contributing to increased crop yields [1]. However, BN residues on agricultural products can adversely affect human bodies [2,3,4], resulting in developmental delays [5], chromosomal changes [6], immune system and neurological issues [7], and endocrine disruptions [8]. Therefore, the development of a sensitive and effective method for detecting trace amounts of pesticides is crucial to ensure human health [9]. In this context, electrochemical sensors have emerged as a prominent tool for rapid molecular detection, offering a combination of swift response times, low cost, and practicality, which are essential for the monitoring of pesticide contaminants [10,11].

Compared to traditional electrochemical detection methods, the electrochemical molecular imprinting technique can achieve highly selective detection of pesticide residues in complex samples [12,13,14,15]. For example, Xue et al. utilized PEDOT-based molecular imprinting polymer (MIP) combined with Co, N co-doped hollow carbon nanotubes for high-performance detection of carbendazim [16]. Ding et al. polymerized o-phenylenediamine on a fluorine-doped tin oxide WO_3_/MoS_2_ for electrochemical impedance sensing of imidacloprid [17]. Mutlu et al. designed MIP based on Co_3_O_4_ for selective detection of deltamethrin [18]. Obviously, the substrate materials play a key role in the sensing performance, where the substrate materials showed a high catalytic effect on these analytes. In addition, it should be noted that, to achieve good conjunctival adhesion of MIP on the substrate material, certain interaction forces should exist between them, such as hydrogen bonding, π-π conjugation, electrostatic interactions, etc. [19]. Thus, choosing a suitable substrate material with customized functionality to modify the electrode surface is beneficial for the film-forming properties of MIP on the electrode.

Currently, metal-organic frameworks (MOFs) have garnered significant interest as functional electrode materials because of their chemical and structural tunability, large surface area, and high porosity, as well as their relatively mild preparation conditions [20,21,22,23,24]. In the same way, MOF-derived carbon materials have also emerged as a research hotspot in the field of electrochemical sensors due to their advantages of outstanding conductivity, remarkable porosity, and rich metal active sites [25,26]. However, for pure MOFs, there is an issue of poor electrical conductivity, which hinders electron transfer [27,28,29]; whereas for pure MOF-derived carbon material, weak functional groups on the surface reduce the interaction forces between the materials and the analytes. The combination of MOFs and MOF-derived carbon can achieve complementary advantages and synergistic catalytic effects, but the applications of their composite materials in sensing have not been reported yet.

**Scheme 1:**
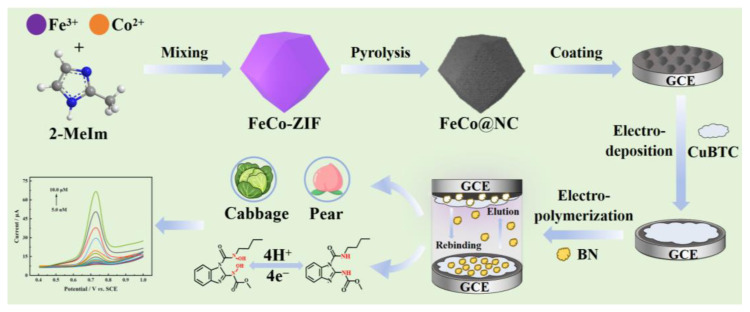
Schematic preparation of PEDOT-MIP/Cu-BTC/FeCo@NC sensing platform and its application for BN detection.

In this study, a new MIP electrochemical sensor was developed based on the composite material of FeCo-ZIF-derived carbon and Cu-BTC. The preparation of the substrate material was first implemented by drop-coating FeCo@NC on the electrode surface, followed by the electrodeposition of Cu-BTC. Subsequently, the molecular imprinted film was prepared on the composite material utilizing the electropolymerization technique (Scheme 1). The Cu-BTC/FeCo@NC composite not only possessed high specific surface area, excellent conductivity, and good catalytic performance, but also provided rich imprinting sites for the formation of stable and dense MIP-PEDOT. The resulting sensor exhibited high electrochemical activity towards BN, demonstrating an enhanced current response. Additionally, this imprinted sensor showed high selectivity, good reproducibility, and reliable performance. Furthermore, satisfactory recovery results were obtained in the determination of BN in cabbage and peaches, indicating the potential of the constructed sensor for food analysis.

## 2. Results and Discussion

### 2.1. Surface Morphology and Structure Characterization

**Figure 1:**
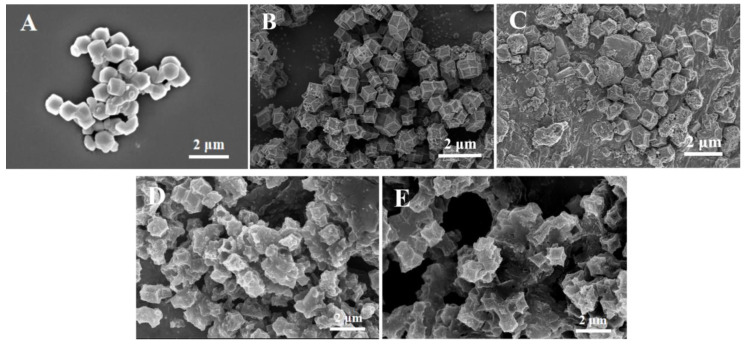
SEM images of FeCo-ZIF (**A**), FeCo@NC (**B**), Cu-BTC/FeCo@NC (**C**), and MIP/Cu-BTC/FeCo@NC before (**D**) and after (**E**) the elution of BN.

The surface morphologies of the electrode materials were observed by scanning electron microscopy (SEM). As shown in Figure 1A, FeCo-ZIF displayed a dodecahedron-shaped morphology with a smooth surface. After pyrolysis treatment, a relatively rough surface with abundant nanoparticles was observed on FeCo@NC (Figure 1B), which was attributed to the formation of Co and Fe nanoparticles. Figure 1C illustrates the microscopic morphology of the Cu-BTC monolithic deposited phase on the FeCo@NC surface. The Cu-BTC exhibited irregular sheet-like stacking, confirming that the Cu-BTC film grew upwards on the FeCo@NC surface. After electropolymerization of PEDOT, a uniform and smooth molecularly imprinted film was coated on the surface of Cu-BTC/FeCo@NC (Figure 1D). In contrast, the surface of the electrode with the template molecules removed presented a porous and rough structure (Figure 1E).

**Figure 2:**
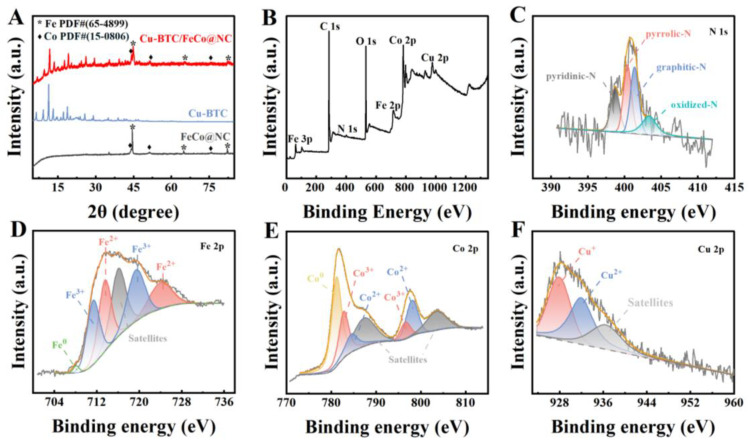
(**A**) XRD curves of FeCo@NC, CuBTC, and Cu-BTC/FeCo@NC; (**B**) total XPS profiles of elements distributed on Cu-BTC/FeCo@NC; XPS spectra of Cu-BTC/FeCo@NC (**C**) N 1s, (**D**) Fe 2p, (**E**) Co 2p, and (**F**) Cu 2p.

To analyze the crystal structures of FeCo@NC, Cu-BTC, and Cu-BTC/FeCo@NC, X-ray diffraction (XRD) spectroscopy of as-prepared samples was investigated (Figure 2A). In the XRD spectra of FeCo@NC, prominent diffraction peaks at 2θ = 44.66°, 66.02°, and 82.31° were attributed to the (110), (200), and (211) crystal planes of Fe (JCPDS no. 65-4899), respectively. Similarly, characteristic peaks were identified at approximately 2θ = 44.22°, 51.52°, and 75.85°, corresponding to the (111), (200), and (220) facets of the Co (JCPDS no. 15-0806). This phenomenon might be due to the fact that metal ions were reduced to their zero-valent state through the high-temperature pyrolysis, confirming the successful formation of FeCo@NC. The XRD pattern of Cu-BTC was consistent with the results reported in the previous research [30], suggesting high crystallinity of the prepared Cu-BTC. After electrodepositing Cu-BTC, the diffraction peak of Fe shifted to a higher angle, while the other diffraction peaks remained unchanged. This shift could be because the introduction of Cu-BTC altered the lattice structure of Fe. These results indicated that Cu-BTC was successfully loaded on the FeCo@NC.

In the FT-IR spectrum of FeCo-ZIF (Figure S1), the characteristic peak at 424.46 cm^−1^ was ascribed to the inorganic–organic (M-N) structure. This confirmed the formation of a bond between metal ions (Fe^3+^ and Co^2+^) and N atoms [31]. The characteristic peaks occurring in the range of 600–1500 cm^−1^ were associated with the stretching and bending pattern of the imidazole ring, whereas bands at 2926.11 and 3132.4 cm^−1^ corresponded to the stretching vibrations of aromatic and aliphatic C-H bonds in 2-methylimidazole [32,33,34]. However, after carbonization, the characteristic peaks in FeCo-ZIF disappeared, confirming the successful formation of FeCo@NC material [35].

Raman spectroscopy was employed to analyze the carbon structure and defect characteristics, revealing distinct D and G bands. As shown in Figure S2, for non-carbonized FeCo-ZIF, the absence of a detectable Raman signal could be attributed to its non-graphitic structure. The absorption bands between 1000 and 1600 cm^−1^ were attributed to C-H stretching and bending modes of the imidazole ring [36]. In contrast, the Raman spectra of FeCo@NC and Cu-BTC/FeCo@NC displayed prominent D (1350 cm^−1^) and G (1580 cm^−1^) bands, with I_D_/I_G_ ratios of 0.88 and 0.74, respectively. The lower I_D_/I_G_ ratio for Cu-BTC/FeCo@NC suggested a reduced defect density and higher graphitization degree compared to FeCo@NC, likely due to the stabilizing effect of Cu-BTC integration during carbonization [37].

The N_2_ adsorption-desorption isotherm of MIP/Cu-BTC/FeCo@NC was measured to investigate the specific surface area value and pore volume. In Figure S3, the significant decrease in specific surface area (from 1139.58 to 392.7 m^2^/g) and pore volume (from 0.631 to 0.332 cm^3^/g) after calcination could be attributed to the structural reorganization of the porous framework and the formation of more dense carbon structures, leading to reduced porosity [38]. Inversely, Cu-BTC/FeCo@NC displayed a specific surface area of 447.71 m^2^/g and a pore volume of 0.280 cm^3^/g. The incorporation of Cu-BTC may partially block the original pore channels of FeCo@NC during the composite formation process, thereby reducing the overall porosity. The reduction in surface area (169.22 m^2^/g) and porosity (0.226 cm^3^/g) observed in MIP/Cu-BTC/FeCo@NC was likely attributed to part of the pores being covered by the MIP film.

X-ray photoelectron spectroscopy (XPS) was employed to characterize the chemical components and valence states of Cu-BTC/FeCo@NC. As shown in Figure 2B, the XPS survey spectrum of Cu-BTC/FeCo@NC confirmed the presence of C, N, O, Fe, Co, and Cu elements. Figure 2C illustrates the N 1s spectra, where the peaks at 398.4, 400.1, 401.2, and 404.7 eV were attributed to pyridine-N, pyrrolic-N, graphitic-N, and oxidized-N. The peak of Fe 2p_3/2_ (Figure 2D) was classified into four peaks: 711.3 eV, 719.4 eV (Fe^2+^), 713.5 eV, and 724.0 eV (Fe^3+^). The peak at 707.3 eV might be attributed to Fe^0^ [39]. In the spectrum of Co 2p (Figure 2E), two satellite peaks (Co 2p_1/2_: 787.56 eV and Co 2p_3/2_: 803.76 eV) were demonstrated. Co^3+^ was represented by the peaks near 782.80 and 796.56 eV, while Co^2+^ was represented by the peaks at 784.71 and 798.53 eV. Moreover, the peak at 781.2 eV belonged to Co^0^ [40]. As shown in Figure 2F, the high-resolution XPS spectra of Cu 2p were classified into two peaks: 928.0 eV (Cu^+^) and 932.0 eV (Cu^2+^) [41]. These results also proved the successful preparation of Cu-BTC/FeCo@NC.

### 2.2. Electrochemical Characterization

**Figure 3:**
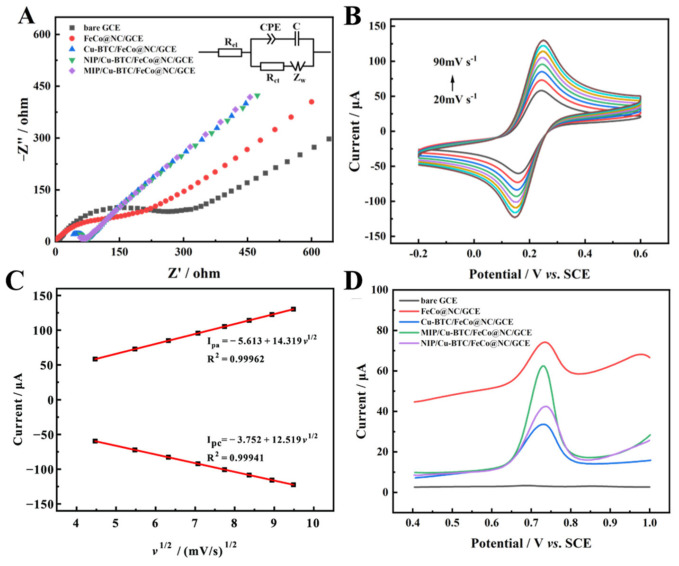
(**A**) EIS plots of electrodes modified with different materials; (**B**) CV curves of MIP/Cu-BTC/FeCo@NC/GCE in 0.1 M KCl containing 5.0 mM [Fe(CN)_6_]^3−/4−^ under different scan rates; (**C**) the linear relationship between *I*_p_ and *v*^1/2^; and (**D**) DPV of bare GCE, FeCo@NC/GCE, Cu-BTC/FeCo@NC/GCE, MIP/Cu-BTC/FeCo@NC/GCE, and NIP/Cu-BTC/FeCo@NC/GCE in 0.1 M PBS (pH 7.0) containing 10 μM BN. Incubation time: 7 min.

The interface electron transfer efficiency of the various electrodes was investigated using electrochemical impedance spectroscopy (EIS). Figure 3A exhibits the EIS plots of bare GCE, FeCo@NC/GCE, Cu-BTC/FeCo@NC/GCE, NIP/Cu-BTC/FeCo@NC/GCE, and MIP/Cu-BTC/FeCo@NC/GCE in 0.1 M KCl solution containing 5.0 mM [Fe(CN)_6_]^3−/4−^. The equivalent circuit parameters derived from the Randles model fitting are summarized in Table S1 [42]. As shown, the excellent electronic conductivity of FeCo@NC gave its charge transfer resistance (*R*_ct_) (247.8 Ω) much lower than bare GCE (370.5 Ω). After deposition of Cu-BTC on FeCo@NC/GCE surface, the *R*_ct_ value (153.6 Ω) of Cu-BTC/FeCo@NC/GCE was decreased. Further assembly of MIP-PEDOT on Cu-BTC/FeCo@NC/GCE made the *R*_ct_ value decrease to 131.5 Ω. These results demonstrated the successful preparation of the MIP/Cu-BTC/FeCo@NC/GCE sensor.

The cyclic voltammetry (CV) curves of MIP/Cu-BTC/FeCo@NC/GCE were measured in 0.1 M KCl solution containing 5.0 mM [Fe(CN)_6_]^3−/4−^ at various scanning rates (Figure 3B). As shown in Figure 3C, the peak current (*I*_p_) increased with the increase in scan rate (*v*), and *I*_p_ and *v* established a good linear relationship. The electrochemical active surface area of MIP/Cu-BTC/FeCo@NC/GCE was calculated through the Randles–Sevcik equation (Equation (1)) [43]:

(1) $$I_{p}=2.69\times 10^{5}n^{3/2}ACD^{1/2}v^{1/2}$$

In which *n* was the number of transferred electrons in the redox reaction of [Fe(CN)_6_]^3−/4−^; *A* represented the electrochemical surface active area; *C* was the concentration of [Fe(CN)_6_]^3−/4−^ (5.0 mM); *D* stood for the diffusion coefficient (7.6 × 10^−6^ cm^2^ s^−1^); and *v* represented the scan rate. The electrochemical active surface area of MIP/Cu-BTC/FeCo@NC/GCE was calculated as 0.1282 cm^2^, which was obviously larger than the bare GCE (0.0707 cm^2^). These results demonstrated that MIP/Cu-BTC/FeCo@NC/GCE had a large active surface area, which facilitated the enrichment of more BN molecules on the electrode surface, thereby amplifying the response signal.

To assess the electrocatalytic characteristics of the modified GCEs, the differential pulse voltammetry (DPV) technique was performed in 0.1 M phosphate buffer solution (PBS) (pH = 7.0) containing 10.0 μM BN. As displayed in Figure 3D, the DPV curve of bare GCE exhibited a weak oxidation peak. Upon the modification of the GCE surface with FeCo@NC, the anodic peak current (*I*_pa_) at 0.736 V exhibited a clear increase. The synergistic effect of Fe and Co and the good electrical conductivity of N-doped carbon resulted in excellent catalytic performance. Further electrodeposition of Cu-BTC on the FeCo@NC surface endowed a much larger oxidation peak current, indicating that the Cu-BTC possessed a high specific surface area and good adsorption capacity towards BN. Following the introduction of the PEDOT-MIP, the peak current at the resulting PEDOT-MIP/Cu-BTC/FeCo@NC/GCE sensor became the largest, which could be attributed to the specific recognition ability of molecularly imprinted films. In contrast, the NIP/Cu-BTC/FeCo@NC/GCE prepared under the same conditions showed a lower current response. The reason might be the fact that the PEDOT-NIP membrane lacks the molecular imprinting cavities on its surface, which are specific recognition sites for the targeted detection of BN.

### 2.3. Optimization of the Experimental Conditions

**Figure 4:**
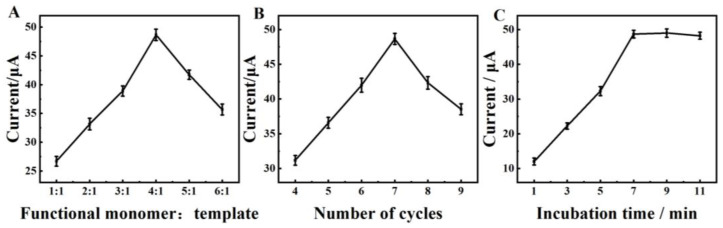
Effect of polymerization cycles of PEDOT (**A**), incubation time (**B**), and the ratio of functional monomer to template molecule (**C**) on current response.

The molar ratio of functional monomers to template molecules was among the factors influencing the performance of the MIP sensor. As shown in Figure 4A, the peak current gradually increased as the molar ratio increased from 1:1 to 4:1 and reached maximum values at 4:1. This may be explained by the functional monomer not being enough to form a polymer with the template if its concentration monomer is low. On the contrary, when the molar ratio further sequentially increased, the current response value decreased. The generation of a thicker imprinting film might reduce the number of imprinting cavities. Therefore, the molar ratio of 4:1 between functional monomers and template molecules was chosen to prepare MIP films.

At the same time, the effect of the number of polymerization cycles on the electrocatalytic activity of BN was investigated. Figure 4B showed that the peak current increased significantly with the increasing number of polymerization cycles, reaching the maximum value at seven cycles. This phenomenon could be explained that with the increase in the number of polymerization cycles, the electrode’s surface was formed with a thicker film, resulting in more imprinted cavities after elution. However, excessive polymerization cycles resulted in overly thick imprinted membranes, which would affect the electron transfer and render many binding sites ineffective. The polymerization of the 7-cycle was selected for further experiments.

Figure 4C exhibited the optimization of incubation time. As time increased from 1 to 7 min, the peak current gradually increased, which can be attributed to the enhanced adsorption capacity of BN molecules onto the MIP/Cu-BTC/FeCo@NC/GCE. The peak current remained stable as the incubation time continued to extend, indicating that the electrode surface adsorption became saturated after 7 min of incubation. Hence, 7 min was chosen as the experimental and real sample analysis incubation time.

**Figure 5:**
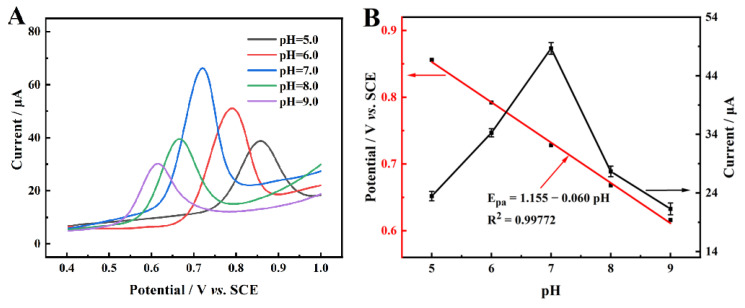
(**A**) DPV of MIP/Cu-BTC/FeCo@NC/GCE in PBS with different pH values containing 10.0 μM BN; (**B**) effect of pH value on the *I*_pa_ and *E*_pa_.

The effect of pH on the electrochemical reaction of MIP/Cu-BTC/FeCo@NC/GCE was investigated with pH ranging from 5.0 to 9.0. As illustrated in Figure 5A, the oxidation peak current increased with the increase in pH values from 5.0 to 7.0, and then decreased at higher pH. The maximum response current was achieved at 7.0. Hence, pH 7.0 PBS was chosen in the following experiments. In addition, as the pH of PBS increased from 5.0 to 9.0, the anodic peak potential (*E*_pa_) of BN shifted negatively, indicating the direct involvement of surface protons in the electrochemical oxidation process of BN. In addition, the peak oxidation potential showed a good linear correlation versus pH as evidenced by the linear regression equation: *E*_pa_ = 1.155–0.060 pH (R^2^ = 0.99772) (Figure 5B). The linear regression slope (60 mV pH^−1^) closely aligned with the theoretical value, indicating that an equal number of protons and electrons were involved in the electrochemical oxidation process of BN occurring on the surface of MIP/Cu-BTC/FeCo@NC/GCE.

### 2.4. Electrochemical Reaction Kinetics

**Figure 6:**
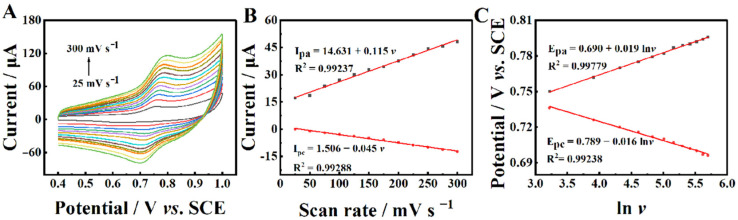
(**A**) CV of 10.0 μM BN on MIP/Cu-BTC/FeCo@NC/GCE in 0.1 M PBS (pH = 7.0) with different scan rates; (**B**) linear relationships of the *I*_pa_ and *I*_pc_ vs. *ν*; and (**C**) the relationships between *E*_pa_ and *E*_pc_ with ln*ν*.

The electrochemical reaction mechanism of BN on the MIP/Cu-BTC/FeCo@NC/GCE surface was investigated by conducting CV in 0.1 M PBS (pH = 7.0) containing 10.0 μM BN at different scan rates. As shown in Figure 6A, the redox peak current gradually increased as the scanning rate increased from 25 mV s^−1^ to 300 mV s^−1^. A minor change was observed in the peak potential. The anodic peak current (*I*_pa_) and the cathodic peak current (*I*_pc_) versus scan rates (*v*) showed clear linear relationships with the linear regression equations of *I*_pa_ = 14.631 + 0.115*v* (R^2^ = 0.99237) and *I*_pc_ = 1.506 − 0.045*v* (R^2^ = 0.99288). These results verified that the electrochemical reaction of BN at MIP/Cu-BTC/FeCo@NC/GCE followed a typical adsorption-controlled process.

In addition, the anodic peak potential (*E*_pa_) and the cathodic peak potential (*E*_pc_) showed a strong linear relationship with the logarithm of the scanning rate in the range of 25 mV s^−1^ to 300 mV s^−1^ (Figure 6B). The linear regression equations can be expressed as *E*_pa_ = 0.690 + 0.019 ln*v* (R^2^ = 0.99779) and *E*_pc_ = 0.789 − 0.016 ln*v* (R^2^ = 0.99238), respectively. According to Laviron’s equation (Equation (2)) [44], the calculated electron transfer number (*n*) was approximately 4. Since the number of protons and electrons in the oxidation process of BN was equal, the oxidation of BN on the electrode surface involved four protons and electrons.

(2) $$E_{pa}=E^{0\prime }+\frac{RT}{(1-\alpha )nF}+\frac{RT}{(1-\alpha )nF}lnv$$

(3) $$E_{pc}=E^{0\prime }+\frac{RT}{\alpha nF}-\frac{RT}{\alpha nF}lnv$$

**Scheme 2:**
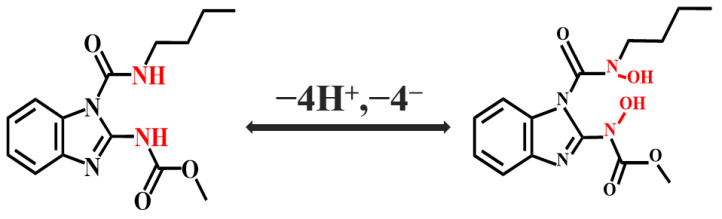
The oxidation mechanism for BN at MIP/Cu-BTC/FeCo@NC/GCE.

Consequently, the electro-oxidation mechanism of BN on the MIP/Cu-BTC/FeCo@NC/GCE is described in Scheme 2.

### 2.5. Electrochemical Detection of BN

**Figure 7:**
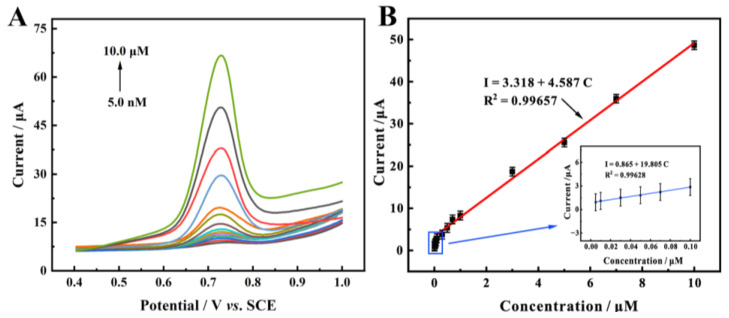
(**A**) DPV curves of the PEDOT-MIP/Cu-BTC/FeCo@NC/GCE in 0.1 M PBS (pH 7.0) with different concentrations of BN (5 nM, 10 nM, 30 nM, 70 nM, 100 nM, 300 nM, 500 nM, 700 nM, 1 μM, 3 μM, 5 μM, 7 μM, 10 μM); and (**B**) linear curve of oxidation peak current vs. concentration of BN.

Under the optimal experimental parameters, the MIP/Cu-BTC/FeCo@NC/GCE sensor was employed for the detection of BN with different concentrations utilizing the DPV method. Figure 7A shows that the oxidation peak currents on the imprinted electrode gradually increased with the increase in BN concentration. A good linear relationship (Figure 7B) was observed between the oxidation peak current and the concentrations of BN. For the low concentration range (5.0 nM–0.10 μM), the linear regression equation was *I* = 0.865 + 19.805C (R^2^ = 0.99628). For the high concentration range (0.3 μM–10 μM), the linear regression equation was *I* = 3.318 + 4.587C (R^2^ = 0.99657). The limit of detection (LOD) was calculated as 1.67 nM (S/N = 3). Furthermore, compared to other electrochemical sensors (Table 1), the MIP/Cu-BTC/FeCo@NC/GCE exhibited a relatively wider linear range and lower LOD.

### 2.6. Reproducibility, Repeatability, Stability, and Anti-Interference Ability

**Figure 8:**
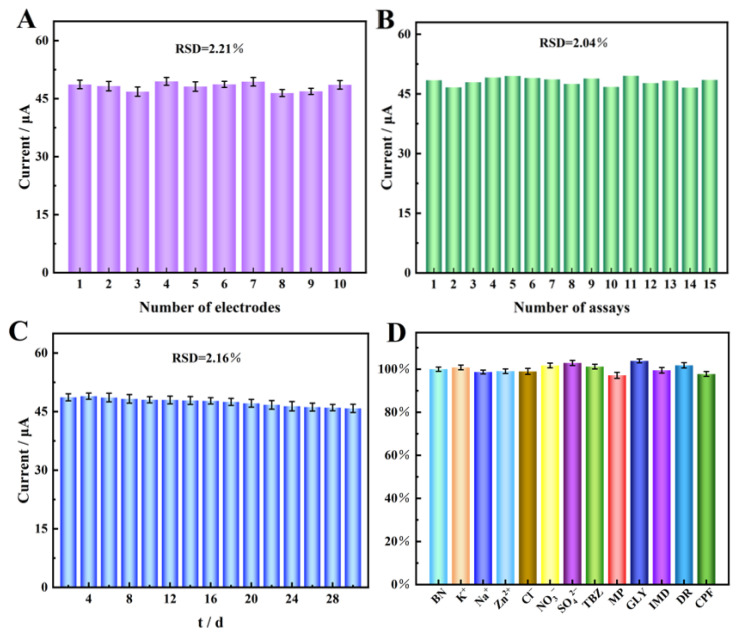
(**A**) Response current of ten parallel MIP/Cu-BTC/FeCo@NC/GCEs toward 10.0 μM BN; (**B**) consecutive detection currents (15 times) of one MIP/Cu-BTC/FeCo@NC/GCE toward 10.0 μM BN; (**C**) detection current of one MIP/Cu-BTC/FeCo@NC/GCE toward 10.0 μM BN within 30 days; and (**D**) influence of 50-fold potential interferences on the response current of 10.0 μM BN.

To evaluate the reproducibility of the sensor, the DPV testing was carried out by using ten modified electrodes in PBS (0.1 M, pH = 7.0) solution containing 10.0 μM BN. As shown in Figure 8A, approximately the same currents were found on the ten with a relative standard deviation (RSD) of 2.21%, suggesting good reproducibility of the sensor. By conducting 15 consecutive tests using one imprinted sensor, the RSD of the test results was calculated as 2.04%, indicating that PEDOT-MIP/Cu-BTC/FeCo@NC/GCE exhibited outstanding repeatability (Figure 8B).

Moreover, to examine the stability, the MIP/Cu-BTC/FeCo@NC/GCE sensor was tested in 10.0 μM BN (0.1 M PBS, pH = 7.0) at two-day intervals for 30 days. After 30 days, the peak current measure could still maintain approximately 94% of the initial current, indicating that PEDOT-MIP/Cu-BTC/FeCo@NC/GCE exhibited good long-term stability (Figure 8C).

Selectivity was an important parameter for assessing the specific recognition capability of molecularly imprinted sensors. The selectivity of the prepared sensor was investigated by adding some potential interfering substances, such as K^+^, Na^+^, Zn^2+^, Cl^−^, NO^3−^, and SO_4_^2−^ ions, thiabendazole (TBZ), methyl-parathion (MP), glycine (GLY), Lambda-cyhalothrin (LMD), atrazine (DR), and chlorpyrifos (CPF) to the BN standard solution. The insecticides and metal ions were included at concentrations 50 times greater than that of BN. Figure 8D showed that the interference of MIP/Cu-BTC/FeCo@NC/GCE to other interfering substances of less than ±5%, indicating the excellent anti-interference ability of the sensor.

### 2.7. Real Samples Detection of BN

The electrochemical detection of BN in cabbage and pear samples was performed with the standard addition method to verify the practicability of the sensor. Cabbage and pear samples were purchased from the local supermarket and diluted in PBS (0.1 M, pH = 7.0). The specific concentration of BN (1.0, 3.0, and 5.0 μM) was separately added to pretreated actual samples, and the detected BN concentrations were determined using the calibration curve in Figure 7B. As shown in Table 2, the recovery rates range from 96.3 to 102.4% (*n* = 5), with calculated RSD values within 4.0%. The results from the investigation suggested the feasibility and accuracy of the MIP/Cu-BTC/FeCo@NC/GCE.

## 3. Experimental Section

### 3.1. Materials

All reagents used in this work were analytical reagent grade and without purification. Co(NO_3_)_2_·6H_2_O and FeCl_3_·6H_2_O were purchased from Aladdin Reagent Co., Ltd. (Shanghai, China). 2-methylimidazole (2-MeIm), pyromellitic acid (H_3_BTC), NaH_2_PO_4_, Na_2_HPO_4_, LiClO_4_, K_3_Fe(CN)_6_, K_4_Fe(CN)_6_, KCl, and methanol were purchased from Vita Reagent Co., Ltd. (Shanghai, China). 3,4-ethylenedioxythiophene (EDOT) and benomyl (BN) were obtained from Macklin Reagent Co., Ltd. (Shanghai, China).

### 3.2. Instruments

Morphology characterization was performed by scanning electron microscopy (SEM, Hitachi S-3400N, Chiyoda City, Japan). The composition and crystal structure of the samples were acquired from X-ray diffraction (XRD, Bruker D8 Advance, Munich, Germany). The elemental compositions and electronic states of the samples were analyzed by X-ray photoelectron spectroscopy (XPS, Thermo Scientific Escalab 250Xi, Waltham, MA, USA). The electrochemical sensing measurements were conducted on a CHI-760E workstation (Shanghai, China) at room temperature.

### 3.3. Synthesis of the FeCo@NC

In a typical synthesis process, Co(NO_3_)_2_·6H_2_O (10 mmol) and FeCl_3_·6H_2_O (0.5 mmol) were dissolved in 120 mL of methanol to form a homogeneous solution. After that, this solvent mixture was added to 120 mL 2-MeIm solution (42 mmol) and stirred magnetically for 24 h. Subsequently, the above solution was maintained at room temperature for 24 h. Purple precipitate was obtained by centrifuging and washing several times with methanol. The product was finally dried overnight at 60 °C in an oven and denoted as FeCo-ZIF.

The FeCo-ZIF sample was then pulverized in an agate mortar and placed into a porcelain boat, which was thermally pyrolyzed in a horizontal tube furnace for 3 h at 800 °C with a constant heating rate of 5 °C min^−1^ under an Ar atmosphere. The obtained product was named FeCo@NC.

### 3.4. Synthesis of Cu-BTC/FeCo@NC/GCE

Before modification, bare GCE was polished to a mirror with 0.05 μm Al_2_O_3_ slurry, followed by ultrasonically washing with ethanol and deionized water for 3 min in turn. Afterward, 2 mg FeCo@NC was dispersed in 1 mL ultrapure water and sonicated for 30 min to form a uniform dispersion. A total of 5 μL of the above suspension was dropped onto the surface of the GCE and dried under an infrared lamp. Afterward, the electrodeposited precursor solution was prepared by dissolving 5 mM CuSO_4_ as a cation source, 15 mM H_3_BTC as the organic linkers in 0.5 mM H_2_SO_4_ under vigorous stirring. Then, the FeCo@NC/GCE was immersed in the precursor solution to serve as the deposition substrate. After 200 s reaction at −1.30 V, the electrode was removed and dried at 40 °C to obtain Cu-BTC films. The obtained electrode was noted as Cu-BTC/FeCo@NC/GCE.

### 3.5. Synthesis of MIP/Cu-BTC/FeCo@NC/GCE

The Cu-BTC/FeCo@NC/GCE electrode was immersed in 5.0 mL phosphate buffer solution (PBS, 0.1 M, pH 7.0) containing 0.01 M EDOT, 0.0025 M BN, and 0.04 M LiClO_4_. The MIP film was electropolymerized using CV with a potential range of 0–1.0 V for 7 cycles at a scan rate of 100 mV·s^−1^. Subsequently, the prepared electrode was placed in a mixed solution containing methanol and acetic acid (9:1, *v*/*v*) for 20 min to remove BN templates from the MIP films. For comparison, the non-imprinted polymers (NIP) were fabricated under the same conditions, except that BN was not added during electropolymerization.

### 3.6. Electrochemical Measurements

The electrochemical measurements were carried out on a CHI760E electrochemical workstation with a three-electrode system. The working electrode, reference electrode, and counter electrode were bare GCE or the modified GCE, a saturated calomel electrode (SCE), and a Pt wire, respectively. CV tests were conducted between −0.2 V and 0.6 V at scan rates ranging from 20 mV to 90 mV in 0.1 M KCl containing 5 mM [Fe(CN)_6_]^3−/4−^ for characterization of the modified electrode surface. The DPV measurements were recorded from −1.0 V to 1.0 V in a solution of 0.1 M PBS (pH = 6.0) containing different concentrations of BN. EIS experiments were performed over a frequency range from 1000 kHz to 0.01 Hz at an amplitude of 5 mV in 0.1 M KCl containing 5 mM [Fe(CN)_6_]^3−/4−^.

### 3.7. Preparation of Real Samples

Cabbage and pear samples were purchased from local supermarkets in Nanchang. A total of 5 g of cabbage and pear samples were cut into small pieces and then mashed in a mortar and filtered through a 0.45 μm membrane. The collected liquid was then centrifuged at 8000 rpm for 10 min, followed by a 100-fold dilution with PBS (0.1 M, pH 7.0). The prepared samples were stored in a refrigerator at 4 °C for further tests.

## 4. Conclusions

In summary, we successfully constructed an MIP/Cu-BTC/FeCo@NC/GCE sensor for the detection of BN in fruits and vegetables. The combination of Cu-BTC MOF and FeCo@NC created a synergistic composite, enhancing the electrochemical oxidation of BN by strengthening the interaction between the material and the target analyte. The stable and dense MIP membrane provided abundant imprinting sites, significantly improving the sensor’s selectivity. The sensor exhibited a wide linear detection range from 5.0 nM to 10.0 μM, a low detection limit of 1.67 nM, and excellent reproducibility, long-term stability, and anti-interference capability. Furthermore, practical sample testing with recovery rates ranging from 96.3% to 102.4% confirmed the sensor’s great potential for real-world applications.

## Figures and Tables

**Table 1:** Comparison of the analytical performance between various reported electrochemical BN sensors.

Sensor	Detection Technology	Sensitivity (μA μM^−1^ cm^−2^)	Peak Potential (V)	Linear Range (μM)	Detection Limit (nM)	References
Bare GCE	adsSWSV ^a^	–	1.09	0.27–5.17	82.66	[45]
OV-17 silicon/CPE	DPV	–	0.67	0.34–13.77	237.66	[46]
NiFe_2_O_4_^b^/MWCNTs/GCE	DPV	–	0.78	0.09–9.98	24.8	[47]
N,P-codoped CNS ^c^	DPV	381.43	0.77	0.025–2	7.50	[48]
MXene-Ti_3_C_2_T_x_/LIG ^d^	DPV	864.3	0.77	0.01–6	5.8	[49]
TiO_2_/TiN@N-C	DPV	8.529	0.74	0.01–1	4.5	[50]
Nano ^e^-Cu_x_O-MWCNTs-COOH/MXene	DPV	70.35	0.76	0.01–10.00	3.0	[51]
ERGO ^f^@ZIF-L ^g^/GCE	DPV	192.92	0.73	0.009–10.00	3.0	[43]
N,P-codoped PCN ^h^	DPV	272.86	0.73	0.005–1.50	2.10	[52]
GDY@PDA ^i^/MWCNTs-NH_2_	DPV	0.45	0.72	0.007–10.00	1.8	[53]
MIP/Cu-BTC/FeCo@NC/GCE	DPV	36.93	0.74	0.005–10.00	1.67	This work

^a^ Square-wave adsorptive stripping voltammetry; ^b^ Magnetic nickel ferrite; ^c^ N-doped carbon nanosheets; ^d^ laser-induced graphene; ^e^ Hierarchical nano; ^f^ Electrochemically reduced graphene oxide; ^g^ Two-dimensional leaf-like framework-L; ^h^ Porous carbon nanosheets; ^i^ Polydopamine.

**Table 2:** Determination of BN in cabbage and pear samples (*n* = 5).

Sample	Added (μM)	Found (μM)	Recovery (%)	RSD (%)
Cabbage	1.0	0.98 ± 0.03	98.0	1.96
	3.0	2.89 ± 0.15	96.3	3.55
	5.0	5.06 ± 0.20	101.2	2.98
Pear	1.0	1.01 ± 0.03	101.0	2.03
	3.0	2.93 ± 0.13	97.7	2.59
	5.0	5.12 ± 0.23	102.4	2.78

## Data Availability

The data presented in this study are available in the article.

## Supplementary Materials

The following supporting information can be downloaded at: https://www.mdpi.com/article/10.3390/molecules30091869/s1, Figure S1. FT-IR spectra of FeCo-ZIF and FeCo@NC. Figure S2. Raman spectra of FeCo-ZIF, FeCo@NC, and Cu-BTC/FeCo@NC. Figure S3. N2 adsorption-desorption isotherms of (A) FeCo-ZIF, (B) FeCo@NC, (C) Cu-BTC/FeCo@NC, and (D) MIP/Cu-BTC/FeCo@NC. Table S1. The parameters of the equivalent circuit. Reference [54] is cited in the supplementary materials.
