# Supplementary material for: Combination of Cu-BTC- and FeCo-MOF-Derived Carbon Enhanced Molecularly Imprinted Electrochemical Sensor for Highly Sensitive and Selective Detection of Benomyl in Fruits and Vegetables
Source: Molecules. 2025 Apr 22;30(9):1869. doi: 10.3390/molecules30091869 (PMC12073791; doi:10.3390/molecules30091869)
Supplement: Supplementary file 1 [file molecules-30-01869-s001.zip › molecules-3520949-supplementary.pdf]

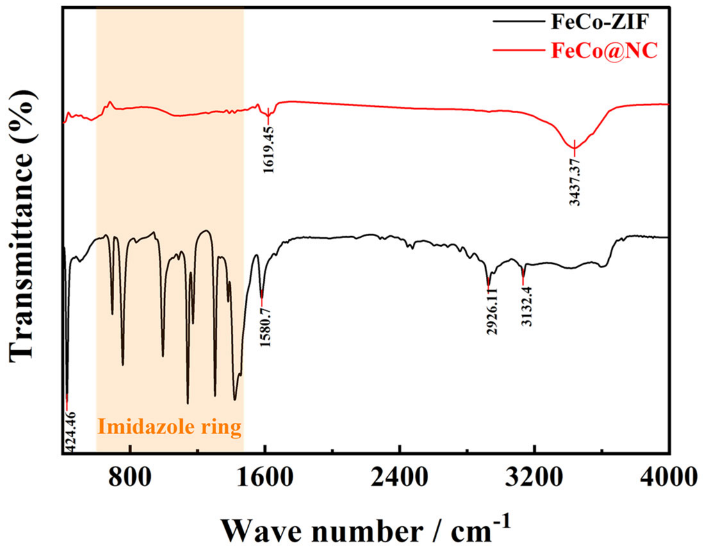


**Figure S1.** FT-IR spectra of FeCo-ZIF and FeCo@NC.

In the FT-IR of FeCo-ZIF (**Figure S1**), the characteristic peak at 424.46 $cm^{-1}$ was ascribed to the inorganic–organic (M-N) structure. This confirmed the formation of a bond between metal ions ($Fe^{3+}$ and $Co^{2+}$) and N atoms [1]. The characteristic peaks occurring in the range of 600–1500 $cm^{-1}$ were associated with the stretching and bending pattern of the imidazole ring, whereas bands at 2926.11 and 3132.4 $cm^{-1}$ corresponded to the stretching vibrations of aromatic and aliphatic C-H bonds in 2-methylimidazole [2-4]. However, after carbonization, the characteristic peaks in FeCo-ZIF disappeared, confirming the successful formation of FeCo@NC material [5].

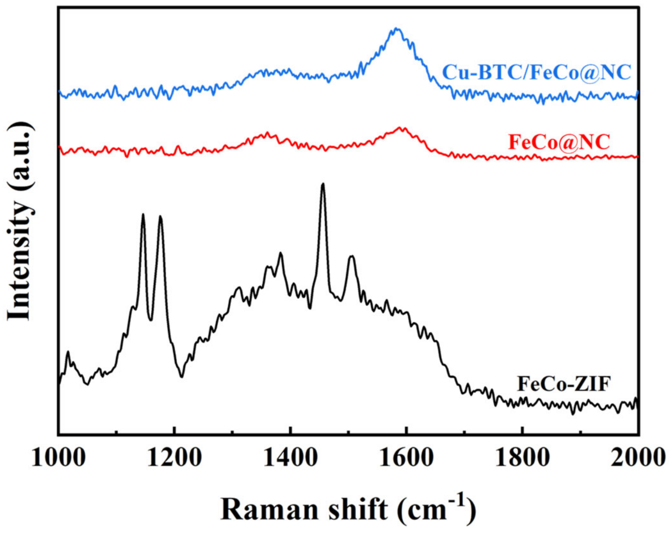


**Figure S2.** Raman spectra of FeCo-ZIF, FeCo@NC, and Cu-BTC/FeCo@NC.

Raman spectroscopy was employed to analyze the carbon structure and defect characteristics, revealing distinct D and G bands. As shown in **Figure S2**, for non-carbonized FeCo-ZIF, the absence of a detectable Raman signal could be attributed to its non-graphitic structure. The absorption bands between 1000-1600 $cm^{-1}$ were attributed to C-H stretching and bending modes of the imidazole ring [6]. In contrast, the Raman spectra of FeCo@NC and Cu-BTC/FeCo@NC displayed prominent D (1350 $cm^{-1}$) and G (1580 $cm^{-1}$) bands, with $I_D/I_G$ ratios of 0.88 and 0.74, respectively. The lower $I_D/I_G$ ratio for Cu-BTC/FeCo@NC suggested a reduced defect density and higher graphitization degree compared to FeCo@NC, likely due to the stabilizing effect of Cu-BTC integration during carbonization [7].

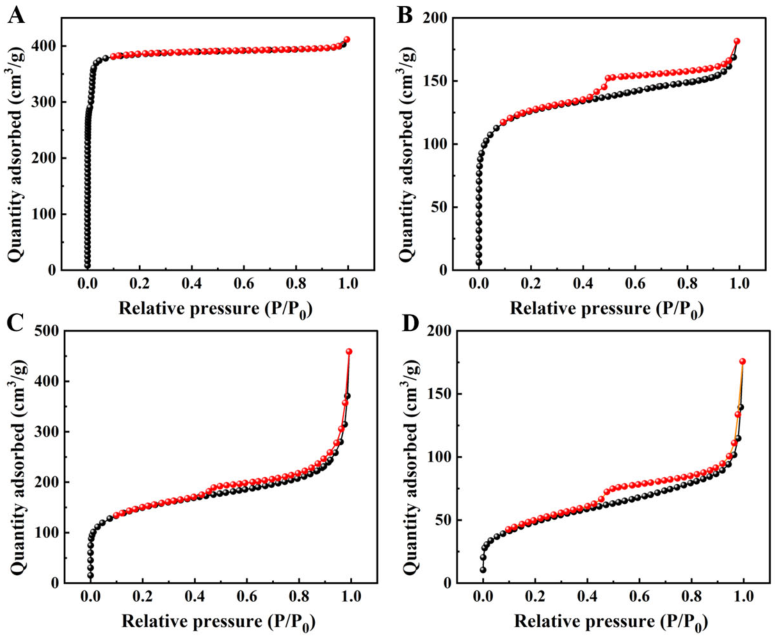


**Figure S3.** $N_2$ adsorption-desorption isotherms of (A) FeCo-ZIF, (B) FeCo@NC, (C) Cu-BTC/FeCo@NC, and (D) MIP/Cu-BTC/FeCo@NC.

The $N_2$ adsorption-desorption isotherm of MIP/Cu-BTC/FeCo@NC was measured to investigate the specific surface area value and pore volume. In Figure S3, the significant decrease in specific surface area (from 1139.58 to 392.7 $m^2/g$) and pore volume (from 0.631 to 0.332 $cm^3/g$) after calcination could be attributed to the structural reorganization of the porous framework and the formation of more dense carbon structures, leading to reduced porosity [8, 9]. Inversely, Cu-BTC/FeCo@NC displayed a specific surface area of 447.71 $m^2/g$ and a pore volume of 0.280 $cm^3/g$. The incorporation of Cu-BTC may partially block the original pore channels of FeCo@NC during the composite formation process, thereby reducing the overall porosity. The reduction in surface area (169.22 $m^2/g$) and porosity (0.226 $cm^3/g$) observed in MIP/Cu-BTC/FeCo@NC was likely attributed to part of the pores being covered by the MIP film.

**Table S1.** The parameters of the equivalent circuit.

| | Electrode | | | | |
|---|---|---|---|---|---|
| | Bare GCE | FeCo@NC/GCE | Cu-BTC/FeCo@NC/GCE | NIP/Cu-BTC/FeCo@NC/GCE | MIP/Cu-BTC/FeCo@NC/GCE |
| $R_{el}$(Ω/cm$^2$) | 135.8±1.77 | 149.7±3.33 | 137.0±1.99 | 99.93±2.24 | 126.8±1.04 |
| $CPE_{dl}$(μS·s$^n$/cm$^2$) | — | — | 68.7±6.09 | 19.0±5.18 | 67.4±16.14 |
| n | — | — | 0.55±0.47 | 0.58±0.19 | 0.54±1.38 |
| $R_{ct}$(Ω/cm$^2$) | 370.5±3.14 | 247.8±2.24 | 153.6±3.27 | 152.9±0.94 | 131.5±5.56 |
| W(mS·s$^{1/2}$/cm$^2$) | 0.40±3.08 | 0.56±3.87 | 0.22±1.91 | 0.70±1.20 | 0.50±1.20 |
| C(μF/cm$^2$) | 0.41 | 0.61 | 0.07 | 2.81 | 6.34 |
| $X^2$ | 0.00488 | 0.00174 | 0.00264 | 0.000865 | 0.00178 |

$R_{el}$: the uncompensated electrolyte solution resistance; $CPE_{dl}$: the constant phase element; n: the roughness factor; $R_{ct}$: the charge transfer resistance; W: the mass transfer resistance; C: the double layer capacitance; $X^2$: weighting factor.

**Reference**


1. Hoang Cao, A. D.; Ha, T. M.; Ngoc Cao, H. L.; Vu Luong, T. H.; Nguyen, T. T.; Chau Nguyen, T. Q.; Dang, G. H. FeCo-ZIFs – Catalyzed indigo carmine removal: A promising approach for wastewater treatment. *Inorg Chem Commun*. **2025,** 173, 113818.
2. Wu, T.; Ma, Z.; Li, P.; Liu, M.; Liu, X.; Li, H.; Zhang, Y.; Yao, S. Colorimetric detection of ascorbic acid and alkaline phosphatase activity based on the novel oxidase mimetic of Fe–Co bimetallic alloy encapsulated porous carbon nanocages. *Talanta.* **2019,** 202, 354-361.
3. Zhang, T.; Ma, Q.; Zhou, M.; Li, C.; Sun, J.; Shi, W.; Ai, S. Degradation of methylene blue by a heterogeneous Fenton reaction catalyzed by $FeCo_2O_4$-N-C nanocomposites derived by ZIFs. *Powder Technol.* **2021,** 383, 212-219.
4. Chen, J.-Y.; Wei, Y.-P.; Chen, J.-S.; Liu, X.-P.; Mao, C.-J.; Jin, B.-K. Self-catalyzed nitrogen-doped carbon nanotubes connected FeCo nanostructures for electrochemical sensitive detection of metol. *Talanta.* **2025,** 290.
5. Wang, Y.; Cao, J.; Yang, Z.; Xiong, W.; Xu, Z.; Song, P.; Jia, M.; Sun, S.; Zhang, Y.; Li, W. Fabricating iron-cobalt layered double hydroxide derived from metal-organic framework for the activation of peroxymonosulfate towards tetracycline degradation. *J Solid State Chem.* **2021,** 294, 121857.
6. Kamali, K.; Prasad, S.; Sahoo, M. K.; Behera, J. N.; Waghmare, U. V.; Narayana, C. Unusual $CO_2$ Adsorption in ZIF-7: Insight from Raman Spectroscopy and Computational Studies. *Inorg Chem.* **2022,** 61, (30), 11571-11580.
7. Zhou, Y.; Wang, J. Degradation of Cephalosporin C using MOF-derived Fe-Co bimetal in carbon cages as electro-Fenton catalyst at natural pH. *Sep Purif Technol.* **2023,** 323, 124388.
8. Pan, L.; Muhammad, T.; Ma, L.; Huang, Z.-F.; Wang, S.; Wang, L.; Zou, J.-J.; Zhang, X. MOF-derived C-doped ZnO prepared via a two-step calcination for efficient photocatalysis. *Appl Catal B-Environ.* **2016,** 189, 181-191.
9. Liangyu Zou, J. X., Qi Liu, Yangqiang Huang, Zhiwu Liang. Strong interaction between efficient magnetic tri-metallic PdFeCo nano-alloy towards formic acid dehydrogenation and application of in-situ hydrogenation. *Int J Hydrogen Energy.* **2024,** 58, (1406), 1417.
